# Nm23-H1-stabilized hnRNPA2/B1 promotes internal ribosomal entry site (IRES)-mediated translation of Sp1 in the lung cancer progression

**DOI:** 10.1038/s41598-017-09558-7

**Published:** 2017-08-22

**Authors:** Chia-Yang Hung, Yi-Chang Wang, Jian-Ying Chuang, Ming-Jer Young, Hungjiun Liaw, Wen-Chang Chang, Jan-Jong Hung

**Affiliations:** 1 Department of Biotechnology and Bioindustry Science, National Cheng Kung University, Tainan, Taiwan; 2 Institute of Basic Medical Sciences, College of Medicine, National Cheng Kung University, Tainan, Taiwan; 3 Center for Infection Disease and Signal Transduction, National Cheng Kung University, Tainan, Taiwan; 4 Graduate Institute of Medical Sciences, College of Medicine, Taipei Medical University, Taipei, Taiwan; 5 The PhD Program for Neural Regenerative Medicine, College of Medical Science and Technology, Taipei Medical University, Taipei, Taiwan; 6 Department of Life Sciences, National Cheng Kung University, Tainan, Taiwan

## Abstract

Our recent studies have indicated that specificity protein-1 (Sp1) accumulates substantially in the early stage of lung cancer but is partially decreased in the late stages, which is an important factor in the progression of the cancer. In this study, we found that Nm23-H1 and hnRNPA2/B1 could be recruited to the 5′UTR of Sp1 mRNA. In investigating the clinical relevance of Nm23-H1/Sp1 levels, we found a positive correlation between lung cancer patients with poor prognosis and low levels of Sp1 and Nm23-H1, suggesting an association between Nm23-H1/Sp1 levels and survival rate. Knockdown of Nm23-H1 inhibits lung cancer growth but increases lung cancer cell malignancy, which could be rescued by overexpression of Sp1, indicating that Nm23-H1-induced Sp1 expression is critical for lung cancer progression. We also found that Nm23-H1 increases the protein stability of hnRNPA2/B1and is thereby co-recruited to the 5′UTR of Sp1 mRNA to regulate cap-independent translational activity. Since the Sp1 level is tightly regulated during lung cancer progression, understanding the molecular mechanisms underlying the regulation by Nm23-H1/hnRNPA2B1 of Sp1 expression in the various stages of lung cancer will be beneficial for lung cancer therapy in the future.

## Introduction

Specificity protein-1 (Sp1) belongs to the specificity protein/Krüppel-like factor (SP/KLF) transcription factor family, and is expressed in mammalian cells^1^. Sp1 is involved in many cellular processes including cell cycle progression, apoptosis, differentiation and tumorigenesis^2–4^. Compared to its expression in normal tissues and cells, the expression of Sp1 is higher in cells and tissues in different cancer types. Sp1 is tightly regulated in the early and late stages of tumorigenesis to influence cancer progression. Studies have indicated that Sp1 is upregulated in the early stage of cancer and downregulated in the late stages^5^. Previous analyses have suggested that transcriptional activity, translational efficacy, and protein stability all contribute to the regulation of Sp1 levels in lung cancer development, indicating that multiple strategies are utilized to control the level of Sp1 during cancer formation^4, 6–8^.

*Nm23*, the first identified metastasis suppressor gene, encodes a multi-functional protein that exhibits anti-metastatic properties^9^. Nm23 has enzymatic and kinase activities including acting as a nucleotide diphosphate kinase, histidine/aspartic acid-specific protein kinase, and serine protein kinase^10, 11^. Downregulation of Nm23 is associated with aggressive behavior in many types of cancer. Recent studies have demonstrated that analysis of Nm23-dependent gene expression is a valid approach for identifying potential modulators of metastatic potential in several cancer types. In addition, Nm23 exhibits an anti-metastatic effect by blocking Ras/ERK signaling^12^. Furthermore, downregulation of Nm23 in pulmonary adenocarcinoma is associated with lymph node metastasis and poor prognosis^13^. Downregulation of PTEN, KAI-1, and Nm23-H1 has been significantly correlated with distant metastasis and is associated with shortened survival in patients with non-small cell lung carcinoma (NSCLC)^14^. Accordingly, recent studies have suggested that analysis of Nm23-H1 as well as FAK protein levels might contribute to predicting the aggressive behavior of NSCLC^15^. Nm23-H1 has been suggested to be an anti-metastasis marker in different cancer types and its expression is related to patient survival. However, regulation of Nm23-H1 and Sp1 in lung cancer progression including proliferation and malignancy, remains unclear.

HnRNPA2/B1 includes two alternative splicing variants-A2 and B1-and belongs to a family of RNA-binding proteins involved in the regulation of gene expression at the transcriptional and translational stages^16^. Overexpression of hnRNPA2/B1 induces neoplastic transformation and might play a role in lung cancer formation^17^. Recent studies have revealed that hnRNPA2/B1 could be an early diagnostic marker for lung cancer detection, implying that hnRNPA2/B1 induction might promote cancer formation^18^. Recent evidence suggests the hnRNPB1variant is more selectively overexpressed than hnRNPA2 in several cancer types, including lung carcinoma, oral squamous carcinoma, oral leukoplakia, and esophageal squamous carcinoma^19–21^. A recent study also indicated that hnRNPA2/B1 modulates the epithelial-mesenchymal transition (EMT) in lung cancer^22^. Therefore, understanding the mechanism underlying the control of hnRNPA2/B1 levels and the role of hnRNPA2/B1 during lung cancer progression is critical for lung cancer prevention. In the present study, we found that the Nm23-H1/hnRNPA2/B1/Sp1 axis might be important for controlling lung cancer progression.

## Results

### Nm23-H1 and hnRNPA2/B1 overexpressed in lung cancer are recruited to the 5′UTR of Sp1 mRNA

**Figure 1:**
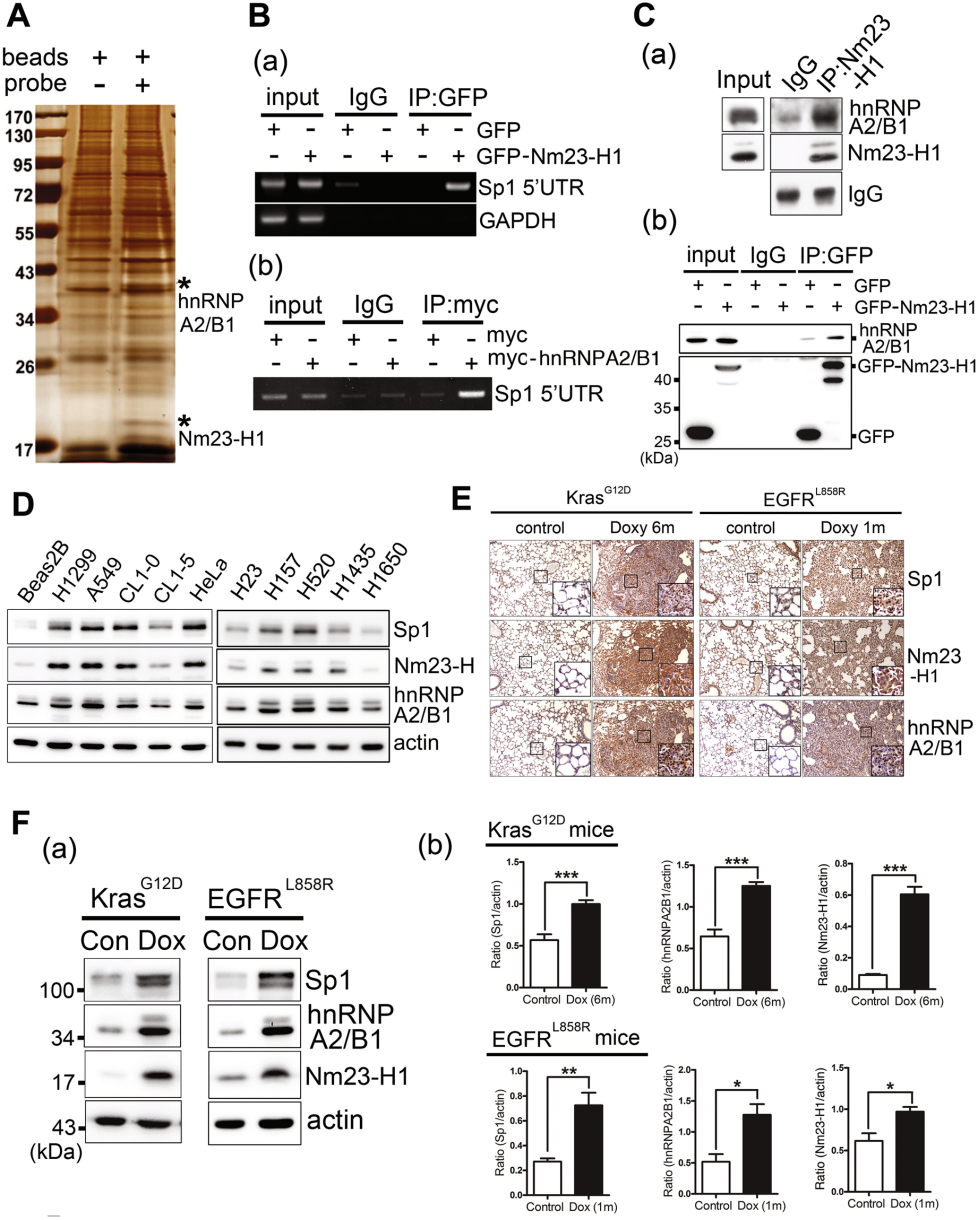
Nm23-H1 and hnRNPA2/B1 overexpressed in lung cancer are recruited to the 5′-UTR of *Sp1* mRNA. (**A**) *In vitro* transcribed pGEM-Sp1-5′-UTR RNA was used as a probe and was incubated with the total cell lysates of HeLa cells. The pulldown mixtures were analyzed using sliver staining and LC MS/MS. (**B**) Samples were harvested from cells with GFP-Nm23-H1 or myc-hnRNPA2/B1 overexpression for IP with antibodies against GFP or myc. RNA fragments were isolated from IP samples for RT-PCR. (**C**) Samples were collected from cells with GFP or GFP-Nm23-H1 expression for IP with anti-Nm23-H1 (a) or anti-GFP (b). IP samples were analyzed by Western blotting with antibodies against the indicated proteins. (**D**) The levels of Sp1, Nm23-H1and hnRNPA2/B1 in the various cancer cell lines were studied by Western blotting with antibodies against indicated proteins. (**E**,**F**) The levels of Sp1, Nm23-H1and hnRNPA2/B1 in mice with Kras^G12D^- and EGFR^L858R^-induced lung cancer were studied using IHC staining (**E**) and Western blotting (**F**). After three independent experiments, the levels were quantified and statistically analyzed with t-test, *p < 0.05; **p < 0.01; ***p < 0.005.

Our previous studies indicated that nucleolin can be recruited to the Sp1 5′UTR and enhances translation in a cap-independent manner in tumorigenesis^8^. According to our previous study, nucleolin is induced from the early to late stages of tumorigenesis^8, 23^. However, Sp1 is downregulated at late stage, which has been suggested to promote metastasis^5, 24^, implying that other factor(s) might also be involved in the regulation of Sp1 during the late stages of lung cancer progression. In addition, we also analyzed the mRNA levels of 537 patients with lung cancer adenocarcinoma (Supplementary Fig. 1). The data indicated that there was no significant difference in the mRNA levels between normal and lung cancer patients, implying that other mechanism(s), such as protein stability or translational activity, might be crucial for the regulation of Sp1 during lung cancer progression. To probe the factor(s) involved in the translational activity of Sp1, we utilized pGEM-Sp1-5′UTR RNA transcribed *in vitro* as a probe that was incubated with the cell lysates. The mixtures were analyzed by silver staining and LC/MS (Fig. 1A). The results showed that hnRNPA2/B1 and Nm23-H1 bound to the 5′UTR of Sp1 mRNA (Fig. 1A). To validate the LC/MS/MS result shown in Fig. 1A, GFP-Nm23-H1 and myc-hnRNPA2/B1 were expressed inside cells and RNA-IP was performed (Fig.1B). The data indicated that, indeed, GFP-Nm23-H1 and myc-hnRNPA2/B1 could be recruited to the 5′UTR of Sp1 mRNA. Next, we found that Nm23-H1 can interact with hnRNPA2/B1 (Fig. 1C), implying that these proteins might regulate Sp1 expression during lung cancer progression. To study the relationship between Sp1, Nm23-H1 and hnRNPA2/B1 expression, the proteins levels were studied in several lung cancer cell lines (Fig. 1D) and mice with Kras^G12D^- and EGFR^L858R^-induced lung cancer (Fig. 1E,F). The data indicated that expression of all three proteins was dramatically induced in lung cancer cell lines and mice with lung cancer compared to that in the normal cells and normal mice, suggesting that these three proteins might be involved in lung cancer progression.

**Figure 2:**
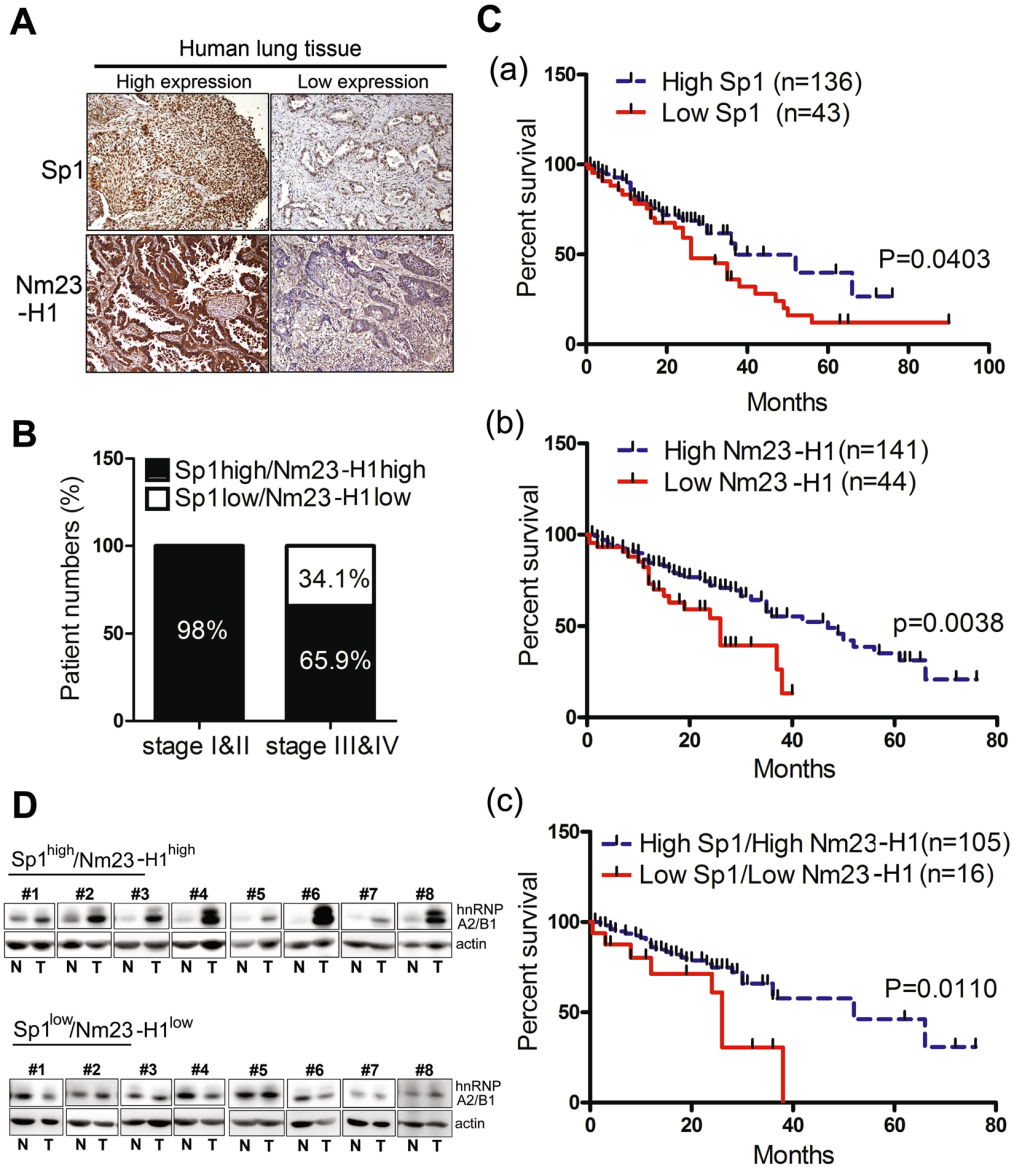
The relevance between Sp1/Nm23-H1 levels and the prognosis of lung cancer patients. (**A**) Samples from lung cancer patients were prepared for IHC staining with antibodies against Sp1 and Nm23-H1. (**B**) After IHC staining samples from 179 patients with anti-Sp1 antibodies and 185 patients with anti-Nm23-H1 antibodies, the relationship between Sp1/Nm23-H1 levels and the stage of lung cancer of the patients was analyzed. (**C**) The survival rate was analyzed between patients with higher Sp1 or lower Sp1 expression (a), patients with higher Nm23-H1 or lower Nm23-H1 expression (b), and between patients with higher Sp1/Nm23-H1 or lower Sp1/Nm23-H1 expression (c) by Kaplan–Meier analysis. The *P*-value was determined by a two-sided log-rank test. (**D**). The level of hnRNPA2/B1 in the clinical samples with high levels of Sp1 and Nm23-H1 or with low levels of Sp1 and Nm23-H1 was studied by Western blotting with anti-hnRNPA2/B1 antibodies.

### Low expression levels of Sp1 and Nm23-H1 are correlated with poor prognosis in patients with lung cancer

Next, the relationship between Sp1/Nm23-H1 expression and the prognosis of patients with lung cancer was addressed (Fig. 2). We collected clinically resected lung cancer specimens for IHC staining. Representative images of low and high expression of Sp1 and Nm23-H1 are shown in Fig. 2A. Of 111 patients with stages I and II lung cancer, 105 (94.6%) exhibited high Sp1 expression, whereas only six (5.4%) had low Sp1 expression. However, of the 66 patients with stage III and IV lung cancer, 30 (45.5%) exhibited high Sp1 expression, whereas 36 (54.5%) showed low Sp1 expression. Furthermore, 141 (76.2%) patients exhibited high Nm23-H1 expression, whereas 44 (33.8%) patients had low Nm23-H1 expression (Table 1). These results indicated that Sp1 and Nm23-H1 were upregulated in the early stages in nearly all of the patients with lung cancer and downregulated in the late stages in certain other patients. Comparison of the expression levels of Sp1 and Nm23-H1 in the different stags demonstrated that Sp1 and Nm23-H1 were highly expressed in most of the patients with stage I and II lung cancer (98%). However, in the late stages, 65.9% of patients were found to exhibit upregulation of Sp1/Nm23-H1, whereas the other 34.1% showed downregulation of Sp1/Nm23-H1 (Fig. 2B). As found in our previous study, low Sp1 was correlated with poor prognosis of lung cancer (Fig. 2C, (a)). Here, we also found that a low level of Nm23-H1 was correlated with a poor prognosis (Fig. 2C, (b)). The correlation of low expression levels of Sp1 and Nm23-H1 with poor prognosis suggested that downregulations of Sp1 and Nm23-H1 promotes lung cancer malignancy (Fig. 2C, (c)). Finally, we also chose 8 samples with high levels of Sp1 and Nm23-H1 and another 8 samples with low levels of Sp1 and Nm23-H1 to investigate the level of hnRNPA2/B1 (Fig. 2D). The data indicated that lung cancer patients with higher levels of Sp1 and Nm23-H1 also exhibited high levels of hnRNPA2/B1, suggesting a positive correlation between the levels of Sp1, Nm23-H1 and hnRNPA2/B1 during lung cancer formation.

**Table 1:** Characteristics of lung cancer patients which are involved in determining Sp1 and Nm23 expression by.

Characteristics	Sp1 high	Sp1 low	*p*-value
**Patients (n)**	135 (76.3%)	42 (23.7%)	
**Nm23 high**	105 (80.2%)	26 (19.8%)	0.0462*^a^
**Nm23 low**	30 (65.2%)	16 (34.8%)	
**Gender** Male	78 (75%)	26 (25%)	0.7208^a^
Female	57 (78.1%)	16 (21.9%)	
**Age (mean ± s.e.m)**	65.3 ± 0.96	65.0 ± 1.6	0.8746^b^
**ADC** ^1^	114 (81.4%)	26 (28.6%)	0.0131*^a^
**SCC** ^2^	17 (58.6%)	12 (41.4%)	
**LCC** ^3^	3 (60%)	2 (40%)	
**Stages I & II**	105 (94.6%)	6 (5.4%)	*p* < 0.0001***^a^
**III & IV**	30 (45.5%)	36 (54.5%)	
**Recurrence**	8 (40%)	12 (60%)	

Abbreviation:^1^adenocarcinoma;^2^squamous cell carcinoma;^3^large cell carcinoma.

^a^Fisher’s extract test, ^b^Studnet’s t test.

### Nm23-H1 regulates Sp1 and hnRNPA2/B1

**Figure 3:**
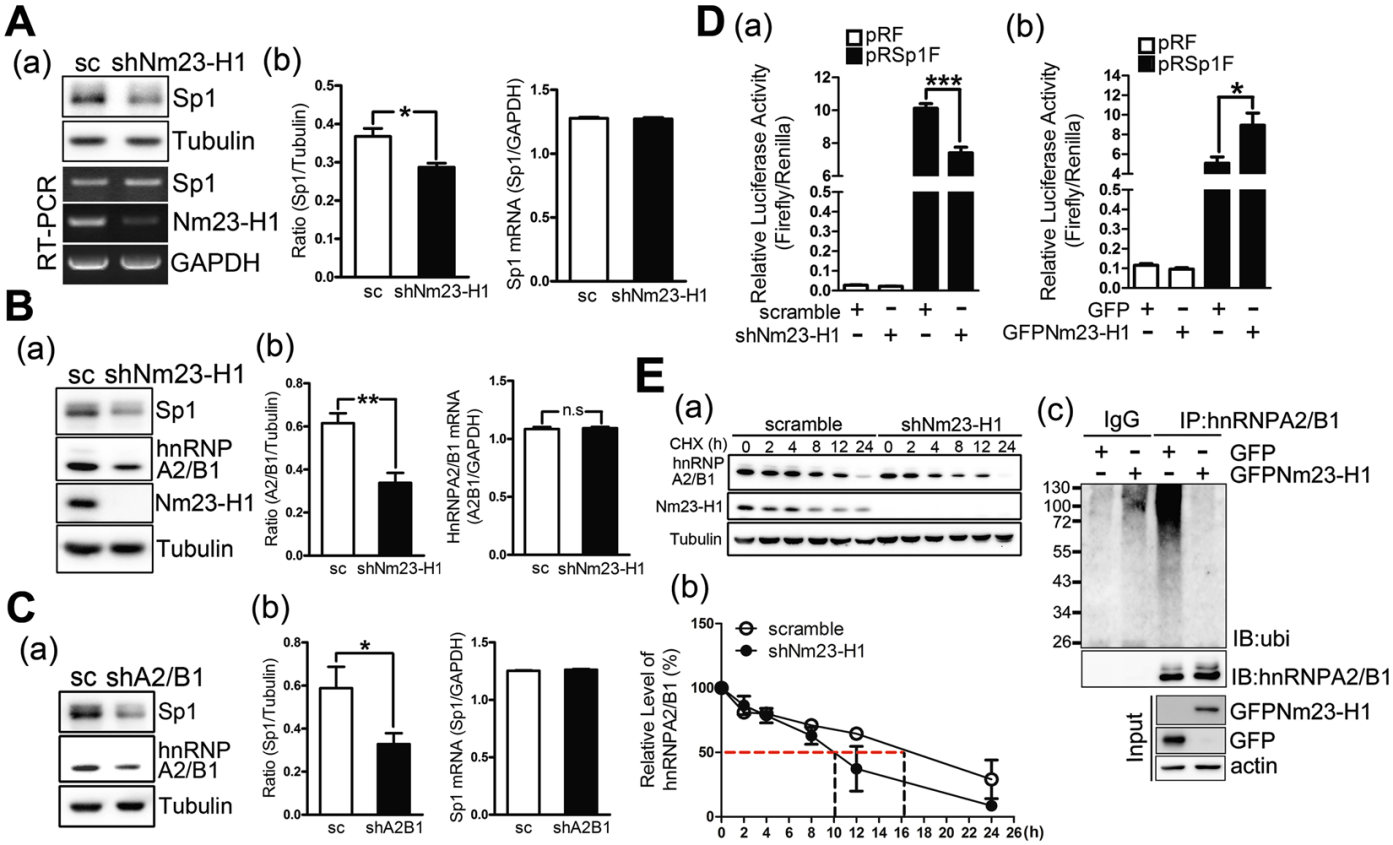
Nm23-H1 regulates Sp1 and hnRNPA2/B1. (**A**–**C**) Nm23-H1 (**A**,**B**) or hnRNPA2/B1 (**C**) was knocked down in CL1-5 cells; thus, the mRNA and protein samples were harvested for real time-PCR and Western blotting with antibodies against indicated proteins. After three independent experiments, the levels of the proteins and mRNA were quantified and statistically analyzed with a t-test, *p < 0.05; **p < 0.01. (**D**) The pRF and pRSp1F plasmids were transferred into lung cancer cells with Nm23-H1 knockdown (a) or GFP-Nm23-H1 overexpression (b). After 24 h of incubation, cell extracts were collected for a luciferase activity assay and analyzed with t-test, *p < 0.05; ***p < 0.005. (**E**) Cells with or without knockdown of Nm23-H1 were treated with cyclohexmide. Samples harvested at the indicated time points were analyzed by Western blotting with antibodies against hnRNPA2/B1, Nm23-H1 and tubulin (a). After three independent experiments, the protein level of hnRNPA2/B1 was quantified (b). Samples were harvested from H1299 cells with or without GFP-Nm23-H1 expression treated MG132 (10 μM) for IP with anti-hnRNPA2/B1 antibodies, and then samples were analyzed by Immunoblotting assay with indicated antibodies (c).

Since Nm23-H1 has been considered as a tumor metastasis suppressor, first, we studied the role of Nm23-H1 in the regulation of Sp1 and hnRNPA2/B1 levels (Fig. 3A,B). The data indicated that knockdown of Nm23-H1 decreased the levels of Sp1 and hnRNPA2/B1 but did not change the levels of *Sp1* and *hnRNPA2/B1* mRNA (Fig. 3A (b),B (b)). In addition, knockdown of hnRNPA2/B1 also decreased the Sp1 protein level but did not change its mRNA level, implying that Nm23-H1 might regulate Sp1 expression through modulating the hnRNPA2/B1 protein level (Fig. 3C). Our recent study showed that an IRES-motif localized within the 5′-UTR of Sp1 mRNA contributed to its translational activity in a cap-independent manner^8, 25^. Therefore, here we studied the effect of Nm23-H1 on the IRES-mediated translational activity of Sp1 (Fig. 3D). The results indicated that knockdown of Nm23-H1 decreased the luciferase activity driven by the 5′-UTR of Sp1 mRNA. Conversely, overexpression of GFP-Nm23-H1 increased the luciferase activity, suggesting that Nm23-H1 positively regulates Sp1 expression through an increase in its translational activity in a cap-independent manner (Fig. 3D). Finally, we found that knockdown of Nm23-H1 decreased the protein stability of hnRNPA2/B1 and overexpression of GFP-Nm23-H1 significantly decreased the ubiquitination signal of hnRNPA2/B1, suggesting that Nm23-H1-induced IRES-mediated translational activity of Sp1 might be through an increase in the protein stability of hnRNPA2/B1 in a proteasome-dependent manner (Fig. 3E).

### Nm23-H1-mediated Sp1 expression is involved in cell growth and the epithelial-mesenchymal transition (EMT) of lung cancer cells

**Figure 4:**
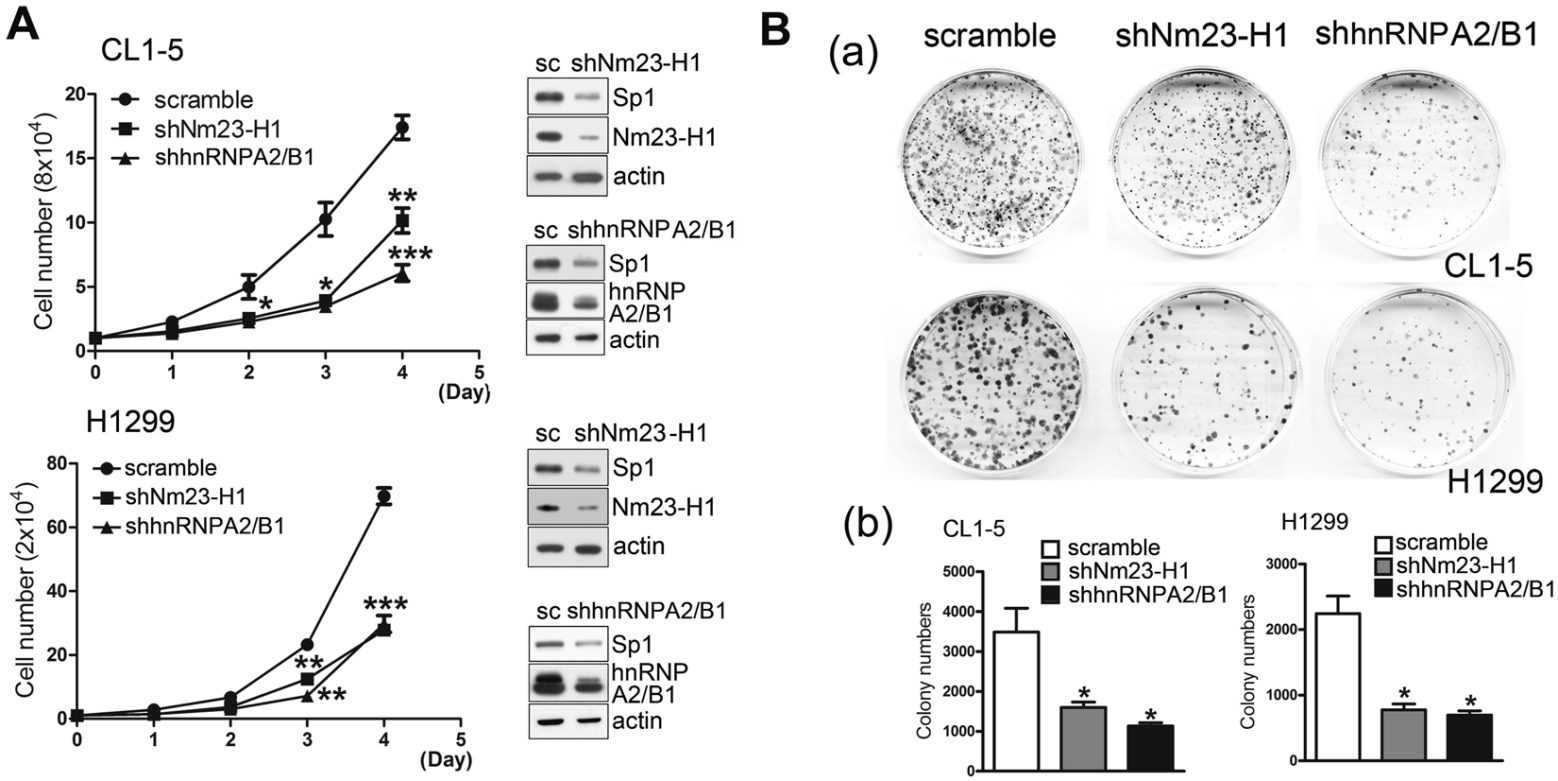
Nm23-H1 and hnRNPA2/B1 increase cell proliferation. (**A**,**B**) Nm23-H1 and hnRNPA2/B1 were silenced in CL1-5 and H1299 cell lines, respectively, for cell counting (**A**) and a colony-formation assay (**B**). After three independent experiments, the levels were quantified and statistically analysis with a t-test, *p < 0.05; **p < 0.01; ***p < 0.005.

**Figure 5:**
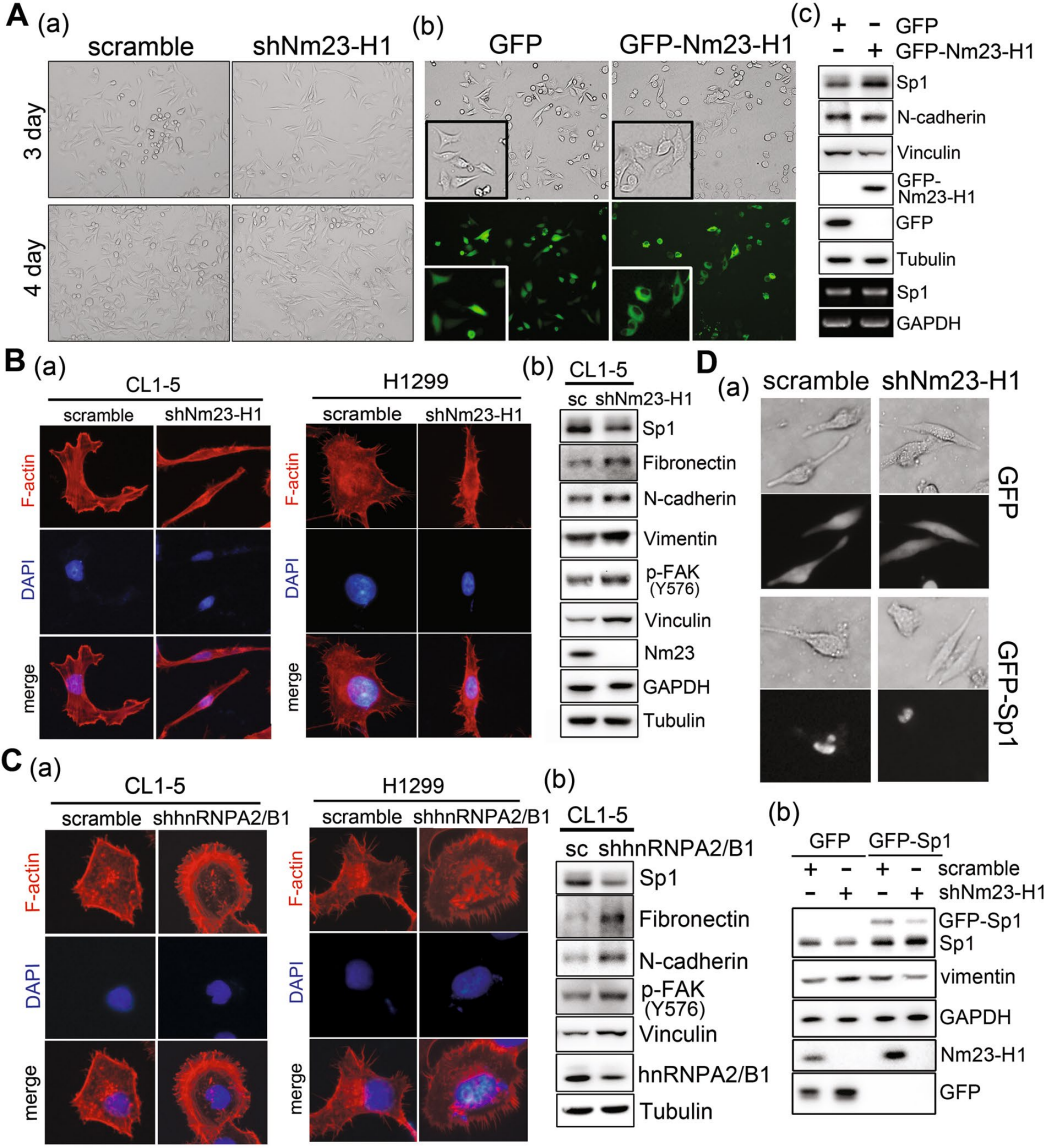
The role of Nm23-H1-mediated Sp1 expression in the epithelial and mesenchymal transition of lung cancer cells. (**A**) Morphology of CL1-5 cells with Nm23-H1 knockdown (a) or overexpression (b) was observed with a microscope (20x), and the samples harvested from GFP-Nm23-H1-over expressed cells were analyzed by Western blotting with antibodies against the indicated proteins (c). (**B**,**C**) The distribution of F-actin in the knockdown Nm23-H1 (**B**) or hnRNPA2/B1 (**C**) in CL1-5 and H1299 cell lines was studied by immunostaining with anti-F-actin antibodies (a). The EMT-related proteins were studied by Western blotting with antibodies against the indicated proteins (b). (**D**) Cell morphology was observed in Nm23-H1 knockdown cells with GFP or GFP-Sp1 overexpression (a), and various EMT-related proteins were analyzed by Western blotting with antibodies against the indicated proteins (b).

The effects of Nm23-H1 and hnRNPA2/B1 on the proliferation of lung cancer cell lines were studied (Fig. 4A,B). The data indicated that knockdown of Nm23-H1 and hnRNPA2/B1 decreased the cell number and colony-formation number in CL1-5 and H1299 cells, suggesting that both Nm23-H1 and hnRNPA2/B1 increase lung cancer cell proliferation. Next, we studied cellular morphology and F-actin polymerization to evaluate the effect of Nm23-H1 on the migratory activity of lung cancer cells (Fig. 5A). The results indicated that knockdown of Nm23-H1 altered the cellular morphology from a round to a slender phenotype. By contrast, overexpression of GFP-Nm23-H1 changed the cellular morphology to be more round. In addition, knockdown of Nm23-H1 also altered the polymerization of actin and increased the protein levels of EMT-related proteins, fibronectin, N-cadherin, vimentin, phospho-FAK and vinculin (Fig. 5B), implying that a decrease in Nm23-H1 increases the invasive ability of lung cancer cells. Since Nm23-H1 increases hnRNPA2/B1 protein stability, we also found that knockdown of hnRNPA2/B1 changed the polymerization of actin and increased expression of EMT-related proteins such as fibronectin, N-cadherin, phospho-FAK and vinculin (Fig. 5C). Finally, since Nm23-H1 and hnRNPA2/B1 can increase the level of Sp1, we also found that GFP-Sp1 expressed in Nm23-H1-silenced cells could rescue the cell morphology from a slender to a more rounded phenotype and decreased the level of vimentin (Fig. 5D). In conclusion, Nm23-H1 and hnRNPA2/B1 regulates Sp1 expression together to affect cell growth and the EMT during lung cancer progression.

### Nm23-H1/hnRNPA2/B1-mediated Sp1 expression is involved in lung cancer malignancy

**Figure 6:**
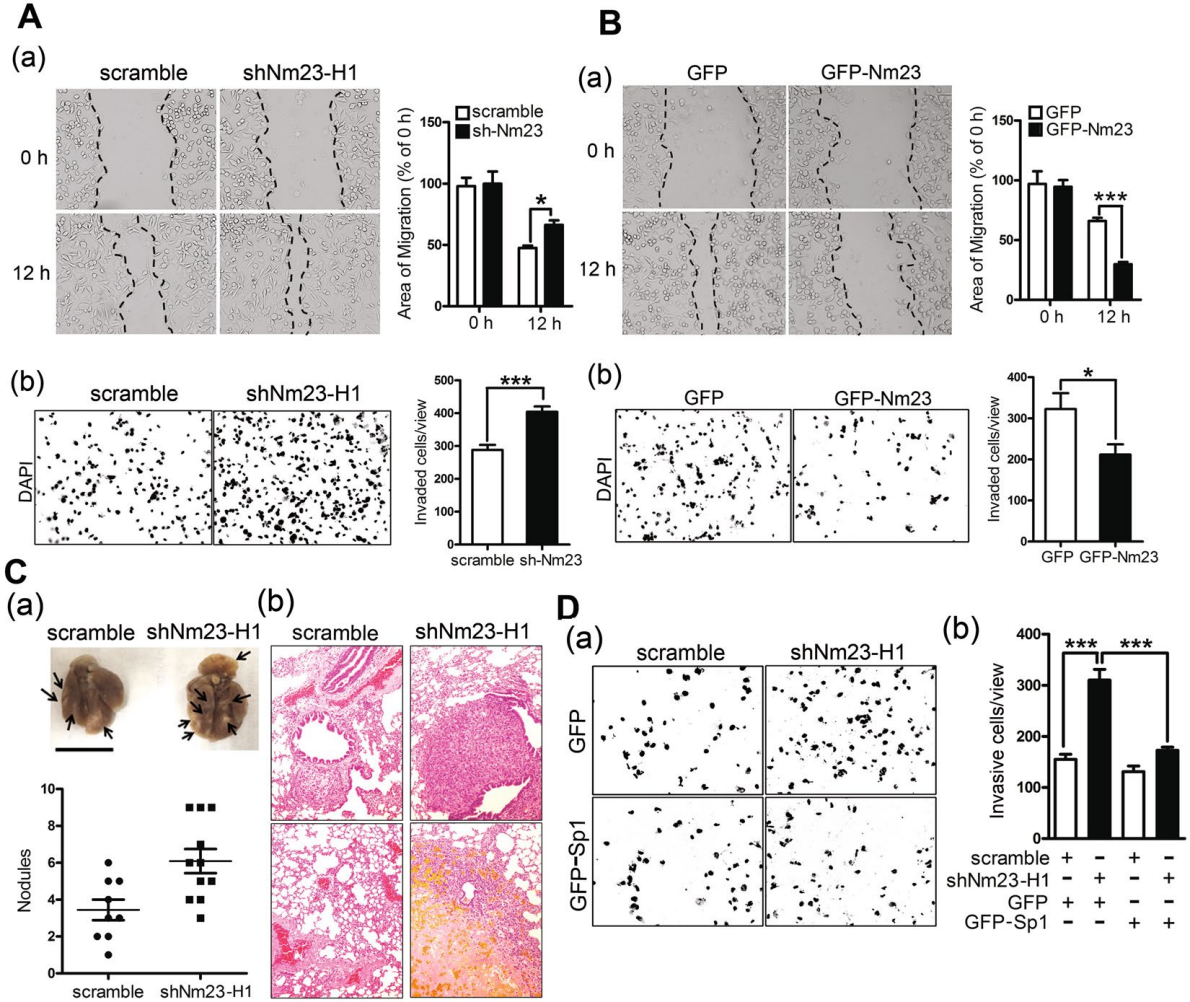
Nm23-H1-mediated Sp1 expression is involved in lung cancer malignancy. (**A**,**B**) The cell migration ability was assayed with a wound healing assay (a) and transwell assay (b) in Nm23-H1 knockdown (**A**) or GFP-Nm23-H1-overexpressed cells (**B**). After three independent experiments, the migratory area and cell number were quantified and statistical analysis was done with a t-test, *p < 0.05; ***p < 0.005. (**C**) CL1-5 cells with Nm23-H1 knockdown were injected into the lateral tail vein of SCID mice. Six weeks later, all mice were sacrificed and the number of tumor nodules was calculated (a). Tumor formation on the lung was studied by H&E staining (b). (**D**) Cell mobility was assayed in the Nm23-H1-silenced cells with or without GFP-Sp1 expression by transwell assay (a). After three independent experiments were finished, the cell number in the chamber was quantified, and the statistical analysis was performed with a t-test, ***p < 0.005 (b).

**Figure 7:**
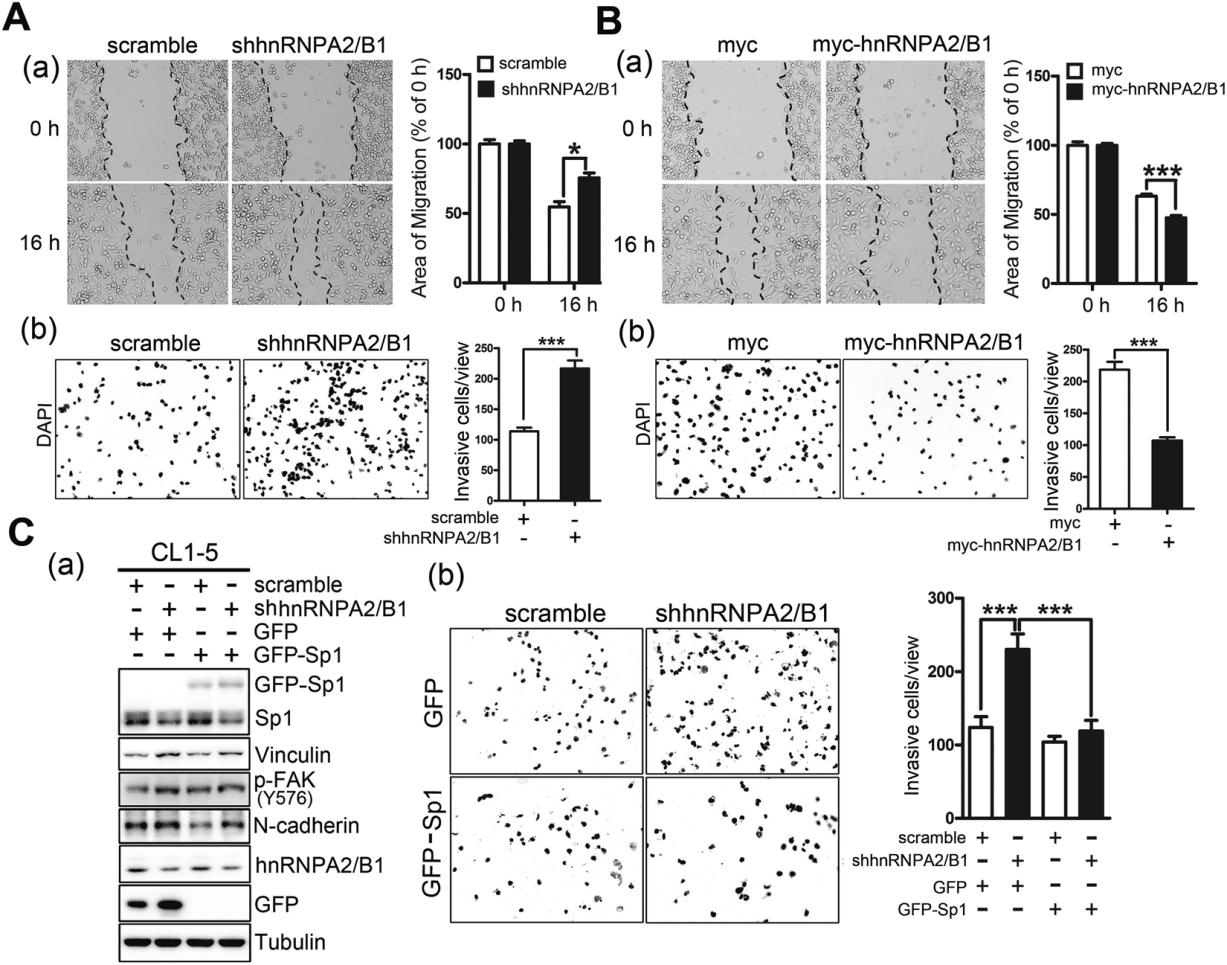
The effect of hnRNPA2/B1 on Sp1-mediated lung cancer migration. (**A**,**B**) Cell mobility was assayed with a wound healing assay (a) and transwell assay (b) in hnRNPA2/B1 knockdown (**A**) or myc-hnRNPA2/B1-overexpressed cells (**B**). After three independent experiments, the migratory area and cell number were quantified, and statistical analysis was done with a t-test, *p < 0.05; ***p < 0.005. (**C**) The protein levels and cell mobility were assayed on the hnRNPA2/B1-silenced cells with or without GFP-Sp1 expression by Western blotting with antibodies against the indicated proteins (a) and by transwell assay (b). After three independent experiments were finished, the cell number on the chamber was quantified and the statistically analysis was performed with t-test, ***p < 0.005.

The previous analyses demonstrated that knocking down Nm23-H1 could change the cellular morphology into that consistent with a more invasive phenotype and inhibited Sp1 in parallel. To determine whether Nm23-H1 could influence cell migration ability, Nm23-H1 was knocked down in CL1-5 cells, and cell mobility was analyzed by wound healing and transwell migration assays. Knockdown of Nm23-H1 increased cell mobility (Fig. 6A), whereas overexpression of GFP-Nm23-H1 inhibited cell migration (Fig. 6B). Furthermore, CL1-5 cells with knocked down Nm23-H1 that were injected into SCID mice formed more lung nodules than control cells injected into mice (Fig. 6C). Finally, we also determined whether Nm23-H1-mediated Sp1 expression could regulate cell migratory ability (Fig. 6D). Knockdown of Nm23-H1 in CL1-5 cells with or without GFP-Sp1 overexpression was assayed using a transwell migration chamber. The results showed that Nm23-H1 knockdown increased cellular migration and that GFP-Sp1 overexpression abolished the effect produced by Nm23-H1 knockdown (Fig. 6D). Based on these results, we concluded that Nm23-H1-mediated Sp1 expression negatively regulates cancer cell migration *in vitro* and *in vivo*. We also examined the role of hnRNPA2/B1 in cellular migration indicated that hnRNPA2/B1 knockdown increased cell migratory activity (Fig. 7A). By contrast, overexpression of myc-hnRNPA2/B1 decreased the migratory activity of lung cancer cells (Fig. 7B). To study the role of Sp1 in hnRNPA2/B1-mediated lung cancer cell migration, GFP-Sp1 was expressed in the cells concomitant with knockdown of hnRNPA2/B1 (Fig. 7C). The results indicated that GFP-Sp1 overexpression could partially reverse elevation of vinculin, phosphor-FAK and N-cadherin levels induced by hnRNPA2/B1 knockdown (Fig. 7C (a)). Overexpression of GFP-Sp1 also almost completely abolished the effect of hnRNPA2/B1 on lung cancer cell migratory activity (Fig. 7C (b)).

### Akt activation is involved in the Nm23-H1-mediated IRES-translational activity of Sp1 mRNA

**Figure 8:**
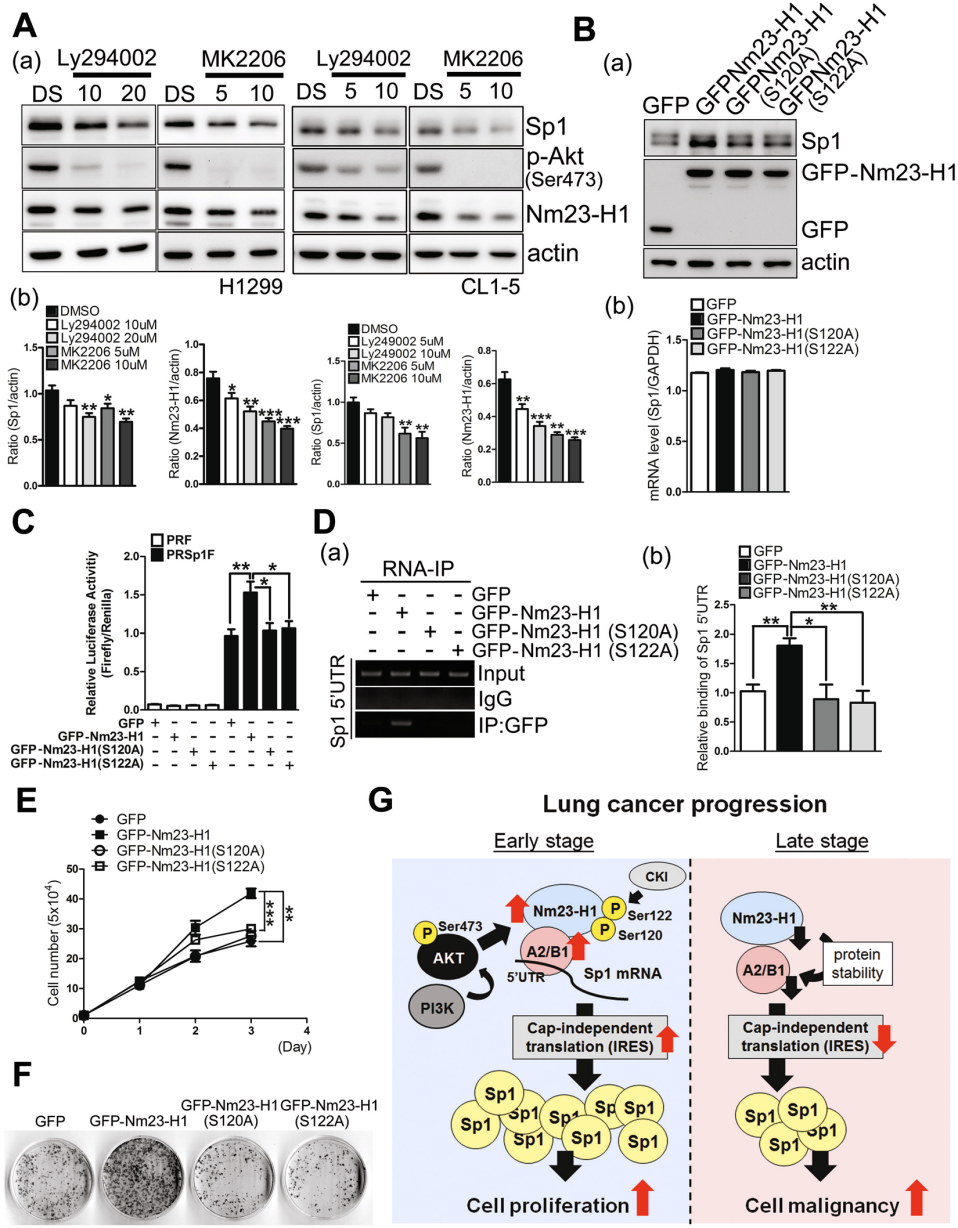
Akt activation might be involved in the Nm23-H1-mediated IRES-translational activity of Sp1 mRNA. (**A**) Samples were harvested from H1299 and CL1-5 cell lines treated with Akt inhibitors, Ly294002 and MK2206, and were analyzed by Western blotting with antibodies against the indicated proteins (a). After three independent experiments, the proteins levels were quantified and statistical analysis was done with a t-test, *p < 0.05; **p < 0.01; ***p < 0.005 (b). (**B**–**F**) Samples harvested from H1299 cells with overexpression of GFP, GFP-Nm23-H1, GFP-Nm23-H1(S120A) or GFP-Nm23-H1(S122A) were analyzed by Western blotting with antibodies against the indicated proteins and real time PCR (**B**), 5′UTR-driven luciferase activity (**C**), RNA-IP with anti-GFP antibodies (**D**), cell counting (**E**) and a colony-formation assay (**F**). After three independent experiments, the proteins levels were quantified and statistical analysis was done with a t-test, *p < 0.05; **p < 0.01; ***p < 0.005. (**G**) The proposed model shows that Nm23-H1 positively regulates Sp1 expression through an increase in its translational activity in a cap-independent manner.

Herein we also found that inhibition of Akt phosphorylation by its inhibitors, Ly294002 and MK2206, in H1299 and CL1-5 cells decreased the levels of Sp1 and Nm23-H1 (Fig. 8A). Previous studies have indicated that Nm23-H1 might be phosphorylated at Ser120 and Ser122 residues^26, 27^. In this study, overexpression of wild-type GFP-Nm23-H1 significantly increased the level of Sp1 but did not affect the mRNA level of Sp1. Overexpression of GFP-Nm23-H1, GFP-Nm23-H1(S120A) and GFP-Nm23-H1(S122A) in H1299 cells with Akt inhibitor, Ly294002, treatment found that inhibition of Akt activity significantly decreased the level of wild-type GFP-Nm23-H1, but just slightly affected the levels of GFP-Nm23-H1 mutants, suggesting that Akt signaling can regulate the level of Nm23-H1 (Supplementary Fig. 2). In addition, this effect in the expression of Sp1 was partly reversed after both of these two phosphorylated resides were mutated (Fig. 8B). Investigating the effect of Nm23-H1 phosphorylation on IRES-mediated translational activity revealed that wild-type GFP-Nm23-H1, indeed, increased the IRES activity of Sp1, but this effect was abolished after loss of the phosphorylation at these two Nm23-H1 sites, indicating that Nm23-H1 phosphorylation at Ser120 and Ser122 is critical for IRES-mediated translational activity (Fig. 8C). Investigating the recruitment of Nm23-H1 to the 5′UTR of Sp1 mRNA showed that wild-type GFP-Nm23-H1 could be recruited to the 5′UTR of Sp1 mRNA, but GFP-Nm23-H1(S120A) and GFP-Nm23-H1(S122A) could not, suggesting that phosphorylation of Nm23-H1 at these two sites is important for the recruitment of Nm23-H1 to the region of the IRES motif located within the 5′UTR of Sp1 mRNA (Fig. 8D). However, the phosphorylation of Nm23-H1 at these two residues did not alter the effect of Nm23-H1 in regulating the level of hnRNPA2/B1, implying that other mechanism(s) of Nm23-H1 regulate the hnRNPA2/B1 level. Finally, the cell and colony number were increased with overexpression of GFP-Nm23-H1 but not with the expression of GFP-Nm23-H1(S120A) and GFP-Nm23-H1(S122A) (Fig. 8E,F). In conclusion, the phosphorylation of Nm23-H1 is essential for the recruitment of Nm23-H1 to the 5′UTR of *Sp1* mRNA to enhance the IRES-mediated translational activity, which might contribute to the proliferation of lung cancer.

## Discussion

Previous studies have shown that regulation of the Sp1 level is critical for lung cancer progression^5, 24^. Therefore, understanding the molecular mechanism(s) of how Sp1 is regulated is important for lung cancer prevention. In this study, we found that Nm23-H1 is increased in the early stages but is decreased in the late stages of lung cancer progression, which is correlated with the Sp1 level. In studying the relationship between Nm23-H1 and Sp1, we found that Nm23-H1-stabilized hnRNPA2/B1 is recruited to the 5′UTR of *Sp1* mRNA, resulting in an increase in its translational activity in a cap-independent manner (Fig. 8G).

Sp1 expression is tightly regulated during cancer progression at the level of protein expression, transactivation activity, and DNA binding affinity^6, 7, 24^. In the early stages of cancer progression, Sp1 is induced to promote cell proliferation^5, 28, 29^. However, during the late stages, Sp1 expression varies in different cancer types. In most cancers such as breast and liver cancers, Sp1 expression remains induced during the late stages and positively regulates cancer malignancy^30, 31^. However, our previous studies indicated that in lung cancer, Sp1 is downregulated during the late stages compared to its expression in the early stages, promoting lung cancer malignancy^5, 24^. In addition to a direct increase in transcriptional activity, many additional strategies for the regulation of Sp1 expression during cancer progression have been reported. For example, previous studies have identified several post-translational modifications of Sp1, which affect its protein stability and result in modulation of Sp1 levels during tumorigenesis^4, 5^. Sumoylation of Sp1 at Lys16 increases the recruitment of RNF4, the Sp1 E3-ligase, resulting in the induction of Sp1 degradation at the late stages of lung cancer progression^6^. Conversely, phosphorylation at Sp1 Thr739 by JNK1 or CDK1 increases its protein stability during the early stages of tumorigenesis. In addition, our recent studies have also indicated that the microRNA miR-182, which is induced in the late stages of lung cancer progression and binds to the 3′UTR of Sp1, could decrease Sp1 translational activity^24^. A recent study also demonstrated that an IRES-motif localized within the 5′UTR of Sp1 mRNA was involved in Sp1 expression during cancer formation^8^. In this study, we used LC/MS/MS to identify the proteins that were bound to the Sp1 IRES-motif, and clarified their effect on Sp1 expression during lung cancer progression. We clearly determined that Nm23-H1-stabilized hnRNPA2/B1 was recruited to the 5′UTR of *Sp1* mRNA to increase its translational activity in a cap-independent manner. It is evident, therefore, based on previous studies and the study presented here, that multiple strategies have been developed by cells to control Sp1 expression during cancer progression. Accordingly, in order to design drugs effective at inhibiting Sp1 expression for use in cancer therapies, the various molecular pathways that contribute to Sp1 expression need to be considered.

Nm23-H1 was the first identified metastasis suppressor gene^9, 10^; however, its function in lung cancer cell growth and the exact molecular process by which Nm23 represses metastasis remain unknown. Knockdown of Nm23-H1 in hepatoma and colon carcinoma cell lines was shown to increase expression of membrane-associated matrix metalloproteinase (MT-MMP) and several pro-invasive signaling pathways such as the Akt and MAPK/SAPK pathways^32^. In addition, Nm23-H1 has been reported to direct dynamin-mediated endocytosis during disassembly of adherin junctions^33^. Some studies have also shown that Nm23-H1/H2 might exert its anti-metastatic effect through blockage of Ras/ERK signaling^34–36^. Previous studies indicated that Nm23-H1 interacts with h-prune and Nm23-H1-h-prune complex positively regulates cell mobility in breast cancer^37^. Nm23-H1-h-prune complex formation was disrupted by CKI inhibitor -IC261 treatment to block the cancer cell mobility. Further reports show that two residues, Ser122 and Ser125, in Nm23-H1 were phosphorylated by CKI to regulate cancer cell progression^26^. However, we found that Akt activation increases Nm23-H1 level. Although Nm23-H1 mutation at these two phosphorylation sites can abolish the effect of Akt activation in Nm23-H1 level, since both of these two sites are not the typical conserve motif, K/R-X-X-T/S, for Akt^38^, we think Akt might not phosphorylate these two residues directly. In this study, we demonstrated a new molecular mechanism by which Nm23-H1 can affect lung cancer progression, wherein Nm23-H1 increases Sp1 expression during lung cancer progression by affecting the IRES-mediated translational activity through stabilizing the protein stability of hnRNPA2/B1; these functions imply that Nm23-H1 upregulation in the early stages of lung cancer and downregulation in the late stages of lung cancer might contribute to the Sp1 level during lung cancer progression. Our previous studies have shown that upregulation of Sp1 increases lung cancer growth and downregulation of Sp1 during the late stages of lung cancer promotes lung cancer malignancy^5, 24^. Herein we found that Nm23-H1 stabilizes hnRNPA2/B1 to increase the IRES-mediated translational activity of Sp1. Akt inhibitor treatment decreased Nm23-H1 expression level that indicated Akt signaling mediated regulation of Nm23-H1. In addition, we also found that Nm23-H1 phosphorylation is important for the IRES-mediated translational activity of *Sp1* mRNA but is not important for hnRNPA2/B1 protein stability, implying that not only hnRNPA2/B1 but also other protein(s) regulated by Nm23-H1 phosphorylation are necessary for the IRES-mediated translational activity of Sp1 mRNA. Finally, Nm23-H1 and hnRNPA2/B1 were present at high levels in nearly all of the examined patients with early-stage lung cancer, indicating that during early-stage lung cancer, the Nm23-H1/hnRNPA2/B1/Sp1 regulatory pathway might promote cancer tumorigenesis by enhancing Sp1 expression. Furthermore, during late-stage lung cancer, a fraction of patients exhibited a decrease in Nm23-H1 expression, whereas the remaining patients showed increases in Nm23-H1 expression; in addition, a poor prognosis was observed for patients with decreased Nm23-H1 levels. Overexpression of Sp1 in Nm23-H1-silenced cells partially abolish the effect of Nm23-H1 on cancer migration, implying that other pathway(s) might also be regulated by Nm23-H1 to affect lung cancer progression.

HnRNPA2/B1 is an RNA-binding protein and has been reported to be overexpressed in various cancer types such as lung cancer at very early stages, suggesting that it might be involved in cancer initiation^20^. A recent study revealed that silencing hnRNPA2/B1 decreased migration in A549 and H1703 cells but not in H358 cells, indicating that the effects of hnRNPA2/B1 on migration in different cancer cell lines can vary considerably^22^. In the present study, we found that hnRNPA2/B1 knockdown increased the migration ability of CL1-5 lung cancer cells, whereas overexpression of myc-hnRNPA2/B1 decreased migration the ability of these cells. We also found that hnRNPA2/B1 could enhance the translational activity of Sp1 via its recruitment to the 5′-UTR of *Sp1* mRNA. Our previous studies have indicated that, in lung cancer, Sp1 downregulation during the late stages of cancer promotes malignancy^5^. A recent study has shown that downregulation of Sp1 during the late stage increased FoxO1 expression, which is an inducer of malignancy, by decreasing the expression of miR-182^24^. Importantly, our study indicated a positive correlation between Sp1 and hnRNPA2/B1 expression during the late stages of lung cancer. These results proved that hnRNPA2/B1, like Sp1, positively regulates cell proliferation but negatively regulates cell malignancy during lung cancer progression. In addition, in mice with Kras^G12D^-and EGFR^L858R^-induced lung cancer, we not only found an increase in hnRNPA2/B1 levels, but also an altered ratio of hnRNPA2/B1 variant expression. However, whether the A2 and B1 variants exert distinguishable roles in modulating lung cancer formation remains to be clarified. We propose two possibilities to explain the effects of hnRNPA2/B1 in cancer malignancy. The first is that hnRNPA2/B1 is an RNA binding protein that regulates multiple cellular functions including translational activity, potentially affecting cancer development in different cancer cell types. The second is that the alteration of the A2 and B1 ratio during cancer progression might be involved in cancer development. However, more experiments need to be performed to clarify this issue. HnRNPA2/B1 has been reported to be one of the early diagnostic markers for cancer prediction^18, 39–41^. Our previous studies have shown that Sp1 is induced in the early period of cancer formation^5^, implying that Nm23-H1, hnRNPA2/B1, and Sp1 could all be considered early biomarkers of lung cancer and should be evaluated for lung cancer prognosis. In conclusion, in this study, we provided evidence to support a role for the Nm23-H1/hnRNPA2/B1/Sp1 axis in the regulation of lung cancer progression; these findings will contribute to a better understanding of lung cancer progression.

## Methods

### Cell culture and transfection

Human lung cancer cell lines, H1299 and CL1-5 were purchased from American Type Culture Collection (Manassas, VA, USA). H1299 and CL1-5 cell lines were cultured in Dulbecco’s modified Eagle’s medium (DMEM) containing 10% fetal bovine serum, 100 mg/ml streptomycin sulfate and 100 U/ml penicillin G sodium at 37 °C and 5% CO_2_ Transfection with indicated plasmid was performed using PolyJet (SignaGen Laboratories, Rockville, MD, USA) and Lipofectamine^TM^2000 (Invitrogen) according to the manufacture’s instruction with a slight modification.

### Western blotting

H1299 and CL1-5 cell lysates were fractionated using SDS-PAGE and transferred onto a PVDF (Millipore, Bedford, MA, USA) membrane using a transfer apparatus according to the manufacturer’s protocols (Bio-Rad Laboratories, Inc., Hercules, CA). After incubation with 5% non-fat milk in TBST (10 mM Tris. pH 8.0, 150 mM NaCl, and 0.05% Tween 20) for 1 h, the membranes were incubated with anti-Sp1 (Millipore), anti-Nm23-H1 (Novus Biologicals, USA), anti-fibronectin, anti-phosphor-FAK, anti-GFP (Santa Cruz Biotechnology), anti-N-cadherin, anti-hnRNPA2/B1, anti-vimentin (GeneTex, CA, USA), anti-vinculin, and anti-tubulin (Sigma) antibody at 4 °C overnight. The membranes were then washed with TBST and incubated with secondary antibodies (Millipore) at room temperature for 1 h blots. After washing with TBST, blots were developed using the ECL system.

### Transwell migration assay

The cell migration assay was performed using Transwell system with an 8-mM pore size polycarbonate filter membrane. After Knockdown of Nm23-H1 or hnRNPA2/B1 in CL1-5 cells for three days and overexpression of GFP-Nm23-H1 or myc-hnRNPA2/B1 in CL1-5 cells for 24 h, cells were trypsinized and suspended in serum-free DMEM. Upper wells were filled with cell suspensions (2 × 10^4^) in serum-free DMEM and lower wells were filled with DMEM containing 10% fetal bovine serum. After incubation for 12 h at 37 °C, the lower side of filter membrane was fixed with 10% formaldehyde and stained with DAPI for 3 min. The migrated cells were counted under a fluorescent microscope.

### Animal experiments

The experiments related with animals were approved by the Institutional Animal Care and Use Committee (IACUC) at National Cheng Kung University (NCKU). Scgb1a1-rtTA/TetO-Kras4b^G12D^ transgenic mice were generated as described previously^5^. Both of Scgb1a1-rtTA and TetO-EGFR^L858R^ mice were purchased from Jackson Lab (Bar Harbor, MA, USA). After breeding, Scgb1a1-rtTA/TetO-EGFR^L858R^ mice were used to study lung cancer. To induce lung cancer progression, two month-old of transgenic mice were orally administrated with doxycycline dissolved in RO water (0.5 g/liter). For drug administration, Scgb1a1-rtTA/TetO-Kras4b^G12D^ mice receiving doxycycline for 5-6 months were sacrificed lung tissues were analyzed by immunohistochemistry staining. Scgb1a1-rtTA/TetO-EGFR^L858R^ mice receiving doxycycline for 4 weeks were sacrificed and lung tissues were analyzed by immunohistochemistry staining. All methods involving animals were performed in accordance with the relevant guidelines and regulations.

### Histological analysis and immunohistochemistry

Lungs excised from Scgb1a1-rtTA/TetO-Kras4b^G12D^ mice, Scgb1a1-rtTA/TetO-EGFR^L858R^ mice, and clinically resected specimens were fixed in 10% formaldehyde for 24 h, dehydrated and embedded in paraffin. Sections (5 μm) were cut and stained with hematoxylin and eosin. For immunohistochemistry, sections were dewaxed in xylene and rehydrated in a graded series of ethanol. Endogenous peroxidases were blocked by 0.3% hydrogen peroxide in phosphate-buffered saline (PBS) for 30 min. Histological sections, which was blocked by 10% bovine serum albumin in PBS, were incubated with appropriate diluted primary antibody for 2 h at room temperature. The immunoreactivity was visualized with a Vectastain ABC kit (Vector Laboratories, Burlingame, CA, USA) and photographed under Olympus BX-51 microscope (Olympus, Melville, NY, USA).

### Collection of clinical specimens from lung cancer patients

Clinical specimens were collected after clinical resection from 190 patients with lung cancer ranging from stages I, II to IV who underwent surgery at the Department of Surgery of the National Cheng Kung University Hospital. The stage of lung cancer was classified by pathologists. Collection of human specimens conformed to the human ethics and was approved by the Clinical Research Ethics Committee at National Cheng-Kung University Medical Center.

All human study has been approved by the Institutional Review Board (IRB) and waived informed consent by the Institutional Review Board (IRB).

### Study Approval

Human study has been approved by the Institutional Review Board (IRB) on Mar. 05, 2013 and the valid execution date is from Aug. 01, 2013 to Jul. 31, 2017 (IRB No: A-ER-101-367). The Institutional Review Board of National Cheng Kung University Hospital (NCKUH) is organized and operated according to the laws and regulations of ICH-GCP. Animal study has been approved by Institutional Animal Care and Use Committee (IACUC) and the valid execution date is from Aug. 01, 2016 to Jul. 31, 2019 (IACUC Approval No: 105217).

### Statistical analysis

Student’s t-test was used to analyze the difference between two groups. Survival curves were calculated according to the Kaplan–Meier method and comparison of survival rate was performed using log-rank test. The *P*-value of 0.05 was considered statistically significant.

## Electronic supplementary material

Supplementary information
