# Supplementary material for: Nm23-H1-stabilized hnRNPA2/B1 promotes internal ribosomal entry site (IRES)-mediated translation of Sp1 in the lung cancer progression
Source: Sci Rep. 2017 Aug 22;7:9166. doi: 10.1038/s41598-017-09558-7 (PMC5567229; doi:10.1038/s41598-017-09558-7)
Supplement: Supplementary file 1 — Supplementary information [file 41598_2017_9558_MOESM1_ESM.pdf]

Nm23-H1-stabilized hnRNPA2/B1 promotes internal ribosomal entry site (IRES)-mediated translation of Sp1 in the lung cancer progression

Chia-Yang Hung [2, 4], Yi-Chang Wang [2], Jian-Ying Chuang [5], Ming-Jer Young [1, 3], Hungjiun Liaw [6], Wen-Chang Chang [5] and Jan-Jong Hung [1,2,3,4,]*

[1] Department of Biotechnology and Bioindustry Science, National Cheng Kung University, Tainan, Taiwan

[2] Institute of Basic Medical Sciences, College of Medicine, National Cheng Kung University, Tainan, Taiwan

[3] Center for Infection Disease and Signal Transduction, National Cheng Kung University, Tainan, Taiwan

[4] Graduate Institute of Medical Sciences, College of Medicine, Taipei Medical University, Taipei, Taiwan

[5] The PhD Program for Neural Regenerative Medicine, College of Medical Science and Technology, Taipei Medical University, Taipei, Taiwan

[6] Department of Life Sciences, National Cheng Kung University, Tainan, Taiwan

***Corresponding author:** Department of Biotechnology and Bioindustry Science, National Cheng Kung University, Tainan 701, Taiwan; Tel: 886-6-275-7575; Fax: 886-6-274-9296;

E-mail: petehung@mail.ncku.edu.tw

Supplementary Fig.1

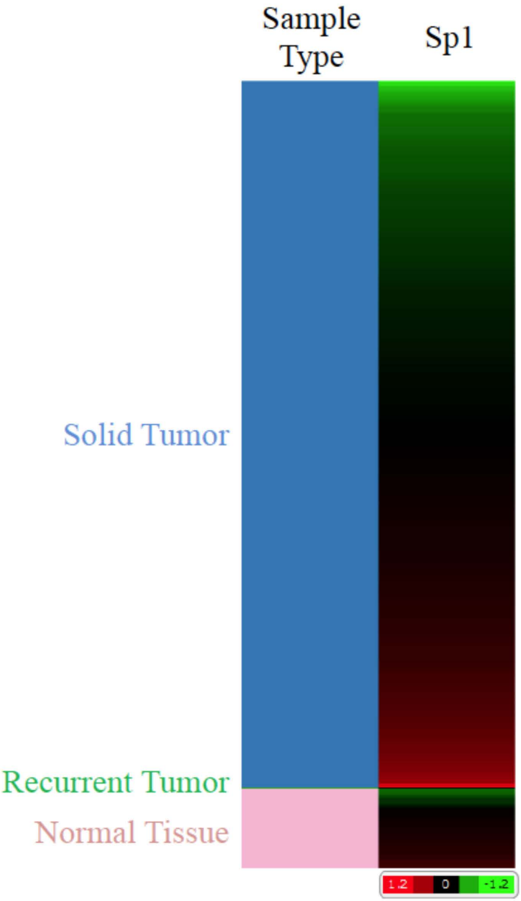

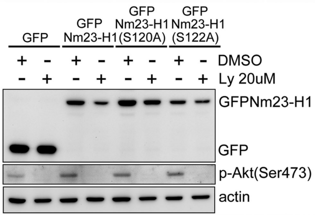

Supplementary Fig.2

Supplementary information

Immunoprecipitation and RNA Immunoprecipitation Assay (RNA-IP)

H1299 and CL1-5 cells were overexpressed GFP, GFP-Nm23-H1, GFP-Nm23-H1 (S120A), GFP-Nm23-H1 (S122A) or myc-hnRNPA2/B1 for 24 h and harvested with lysis buffer (10 mM Tris–HCl, pH 7.4, 150 mM NaCl, 5 mM EDTA, 0.1% sodium dodecyl sulfate (SDS), 1% NP-40, 0.25% deoxychlate, and 1U/μl RNaseOUT) with freshly added protease inhibitor (BioVision, California, USA) for 20 min on ice. The total cell extracts were incubated with anti-GFP (Santa Cruz Biotechnology) or anti-myc (Cell Signaling Technology) at 4°C overnight with rotation. The mixture was incubated with protein A/G agarose beads (Millipore) at 4°C for 2 h with rotation. Immunoprecipitated complexes were washed with lysis buffer and total protein was analyzed by western blot. Total RNA was extracted by TRIzol reagent (Invitrogen) and mRNA was detected by RT-PCR analysis.

In vivo metastasis assay

The animal study was approved by the institutional animal care and use committee at the National Cheng-Kung University. After knockdown of Nm23-H1 in CL1-5 cells for three days, cells were trypsinized and suspended in PBS for tail vein injection. A total of 10^6 cells in 100 ml of PBS were injected into the lateral tail vein of 4-week-old severe combined immunodeficient mice (five mice per group). Mice were killed after 8 weeks and excised lungs were fixed with 10% formaldehyde for 48 h. Finally, the number of metastatic nodules on the surface of lung was counted and lungs were prepared for paraffin sections stained by hematoxylin and eosin.

Immunofluorescent staining

After overexpression of GFP or GFP-Nm23-H1 in CL1-5 cells for 24 h and cells were seeded onto coverslips. Fixation with 4% paraformaldehyde (Sigma) in PBS for 10 min and permeabilization with 0.1% Triton X-100 for 10 min, cells on the coverslip were blocked with 2% bovine serum albumin for 1 h and stained with the antibody against GFP (1:200;

Santa Cruz Biotechnology) and F-actin (1:200; GeneTex) for 1 h at room temperature. Subsequently, cells on the coverslip were washed with PBS three times and stained with the anti-mouse or anti-rabbit antibody (1:200, Jackson ImmunoResearch Laboratories, West Grove, PA, USA) for 1 h at room temperature. Finally, cells were washed with PBS three times and mounted with 90% glycerol containing DAPI (Invitrogen) and photographed under Olympus BX-51 fluorescent microscope (Olympus) at x1000 magnification.

Vitro RNA Synthesis

The plasmid pGEM-Sp1-5′-UTR was linearized to serve as templates to generate RNA probe for pulldown or translational assays. The RNAs were synthesized using the Riboprobe *in vitro* Transcription system (Promega). The linearized DNA template was transcribed by SP6/T7 polymerase in the presence of 2.5 mM UTP, CTP, ATP, and GTP. After 1 h incubation at 37°C, the reaction was stopped by adding 2 U of RQ1 RNase-free DNaseI (Promega) for 15 min at 37°C. The RNA was purified with columns for pull-down assay.

Biotin Pull-Down Assay

The biotin-labeled RNA incubated with HeLa cell lysates for 4 h at 4°C. The RNA-protein complex was isolated by streptavidin-conjugated agarose beads and washed with wash buffer (10 mM HEPES, pH 8.0, 40 mM KCl, 3 mM $MgCl_{2,}$ 5% glycerol, 2 mM DTT, and 2% NP-40) supplemented with protease inhibitors (1 mg/ml leupeptin, 1 mg/ml aprotinin, and 0.5 mM phenylmethylsulfonyl fluoride). The RNA/protein complex was analyzed by SDS-polyacrylamide gel electrophoresis (SDS-PAGE) and silver staining. After silver staining, these specific bands were analyzed by LC MS/MS.

Wound healing assay

Knockdown of Nm23-H1 or hnRNPA2/B1 in CL1-5 cells for three days or overexpression of GFP-Nm23-H1 or myc-hnRNPA2/B1 in CL1-5 cells for 24 h, the linear wound of cellular monolayer was created by scratching confluent cell monolayer using a plastic pipette tip. Scratched cell monolayer was washed by PBS to remove debris. After incubation at 37°C for

12-16 h, area of migration was photographed under light microscope for evaluation.

RNA interference

RNA interference vectors that were used in this study and were obtained from the National RNAi Core Facility in the Institute of Molecular Biology, Academia Sinica (Taipei, Taiwan) are as follows: pLKO.1-shRNA-Nm23-H1 (target sequence, 5′-GCGTACCTTCATTGCGATCAA-3′) and pLKO.1-shRNA-hnRNPA2/B1 (target sequence, 5′- CAGAAATACCATACCATCAAT -3′).

RNA extraction, reverse transcription-polymerase chain reaction (RT-PCR)and real time PCR

Total RNA of H1299 and CL1-5 cells was isolated with TRIzol reagent (Invitrogen, Carlsbad, CA) and 3 μg of RNA was subjected to RT-PCR with SuperScriptII (Invitrogen). The primers used are listed as follows: for Sp1 5′-UTR forward: 5′-ACTAGTAGCGAGTCTTGCCATTGG-3′ and reverse: 5′-GGCGCCGGTGGCAGCTGAGGGACA-3′, Sp1 forward: 5′-TGCAGCAGAATTGAGTCACC-3′ and reverse 5′-CACAACATACTGCCCACCAG-3′ , GAPDH forward: 5′-CCATCACCATCTTCCAGGAG-3′ and reverse: 5′-CCTGCTTCACCACCTTCTTG-3′, Nm23-H1 forward: 5′-ACCTTCATTGCGATCAAACC-3′ and reverse 5′-GGCCCTGAGTGCATGTATTT-3′, hnRNPA2/B1 forward: 5′-GGCTACGGAGGTGGTTATGA-3′ and reverse 5′-ATAACCCCCACTTCCTCCAC-3′.
